# A pivotal role for ocean eddies in the distribution of microbial communities across the Antarctic Circumpolar Current

**DOI:** 10.1371/journal.pone.0183400

**Published:** 2017-08-21

**Authors:** Siddarthan Venkatachalam, Isabelle J. Ansorge, Adriano Mendes, Lebohang I. Melato, Gwynneth F. Matcher, Rosemary A. Dorrington

**Affiliations:** 1 Department of Biochemistry and Microbiology, Rhodes University, Grahamstown, South Africa; 2 Department of Oceanography, Marine Research Institute, University of Cape Town, Rondebosch, South Africa; Laval University, CANADA

## Abstract

Mesoscale variability and associated eddy fluxes play crucial roles in ocean circulation dynamics and the ecology of the upper ocean. In doing so, these features are biologically important, providing a mechanism for the mixing and exchange of nutrients and biota within the ocean. Transient mesoscale eddies in the Southern Ocean are known to relocate zooplankton communities across the Antarctic Circumpolar Current (ACC) and are important foraging grounds for marine top predators. In this study we investigated the role of cyclonic and anti-cyclonic eddies formed at the South-West Indian Ridge on the spatial variability and diversity of microbial communities. We focused on two contrasting adjacent eddies within the Antarctic Polar Frontal Zone to determine how these features may influence the microbial communities within this region. The water masses and microbiota of the two eddies, representative of a cyclonic cold core from the Antarctic zone and an anti-cyclonic warm-core from the Subantarctic zone, were compared. The data reveal that the two eddies entrain distinct microbial communities from their points of origin that are maintained for up to ten months. Our findings highlight the ecological impact that changes, brought by the translocation of eddies across the ACC, have on microbial diversity.

## Introduction

Advances in our methods for identifying and tracking mesoscale eddies across the global oceans have dramatically improved with the development of satellite altimetry and its ability to construct a global atlas of eddy trajectories, amplitudes, and sizes [1,2]. In the past two decades, advances in the theory, observations and eddy-resolving models have resulted in a better understanding of the importance of eddy-induced physical-biological-biogeochemical interactions within the ocean [2]. The physical processes occurring within these ocean eddies (such as eddy stirring, trapping, eddy pumping) play a decisive role in the dynamics of the water column and the depth of the mixed layer and in turn the successful functioning of ecosystems across all trophic levels [2,3]. Eddies thus play an important role in the biogeochemistry and associated microbial and carbon fluxes of the upper ocean [2, 4–6], as they provide contrasting physical environments often within close proximity to one another.

**Fig 1:**
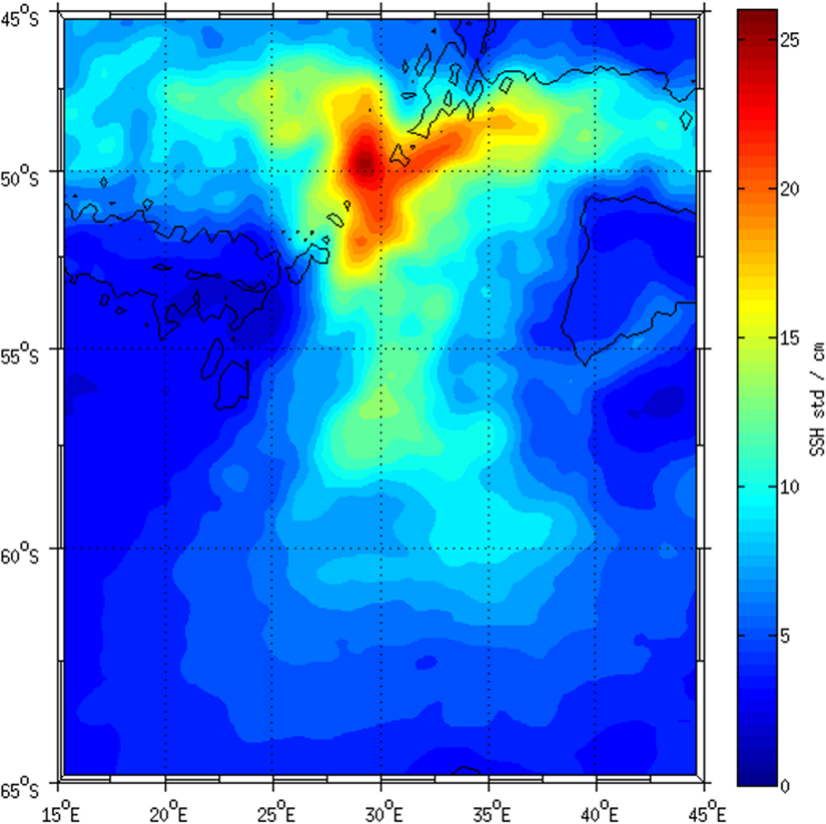
Sea surface height (SSH) variability for the period 2000–2009 for the South-West Indian Ridge (SWIR). The region of interest in this study is the southward extension of variability centred along 30°E from 50–58°S. The black lines represent the 3500 isobath and demarcate the location of the SWIR in relation to this region of high ocean variability.

Within the Southern Ocean, observations [7] have shown that the mean eddy kinetic energy associated with the Antarctic Circumpolar Current (ACC) is almost nonexistent over the deep ocean basins, where topographic constraint is weak, but surges on encountering prominent bathymetric features or choke points such as the Drake Passage [8, 9], the Crozet and Kerguelen Plateaux [10] and south of Australia [11]. One such region is the South-West Indian Ridge [12–14] (Fig 1). This ocean “hotspot” coincides with the southward deflection and intensification of the ACC at 30°E, resulting in an extensive eddy train that extends eastwards towards the Prince Edward Islands at 37°E [15, 16]. This form of oceanic mesoscale and sub-mesoscale instability affects both the lateral advection and the upwelling patterns that influence nutrient and microbial input [2, 17]. Furthermore, these eddies are known to have a significant biological influence [18, 19] by transporting their physical characteristics and biota, typical of either the Antarctic or Subantarctic, across the ACC. Regional investigations in the vicinity of the Prince Edward Islands have shown that eddies provide an important foraging ground for the islands’ resident grey-headed albatrosses [20] and elephant seals [21–23].

Previous studies show that the environment upstream of the Prince Edward Islands is rich in eddies characterized by mixed plankton communities and diverse biogeography [24]. Antarctic, Subantarctic and even Subtropical zooplankton species have been recorded in the vicinity of these islands [25]. An investigation into the biogeography of a cold-core Antarctic eddy revealed significant differences in the macro zooplankton composition both inside and outside of the eddy that could be back-tracked to its point of origin [19]. It is well known that eddies typical of the Subantarctic domain are also spawned by the southward deflection of the ACC in this region, but they are yet to be biologically characterized. Simply put, eddies form turbulent ocean whirlwinds that, depending on their rotation have the ability to stimulate or suppress surface layers of their nutrient and microbial content.

The opportunity to compare the impact of contrasting ocean dynamics on the microbial communities of Southern Ocean eddies presented itself between 22 April and 7 May 2012. The objective of the 2012 survey was to investigate the exact nature of two contrasting, adjacent eddies within this “eddy hotspot” (Fig 1) and to determine how these features influence the microbial communities within the Antarctic Polar Frontal Zone (APFZ).

## Materials and methods

### Oceanographic data collection

**Fig 2:**
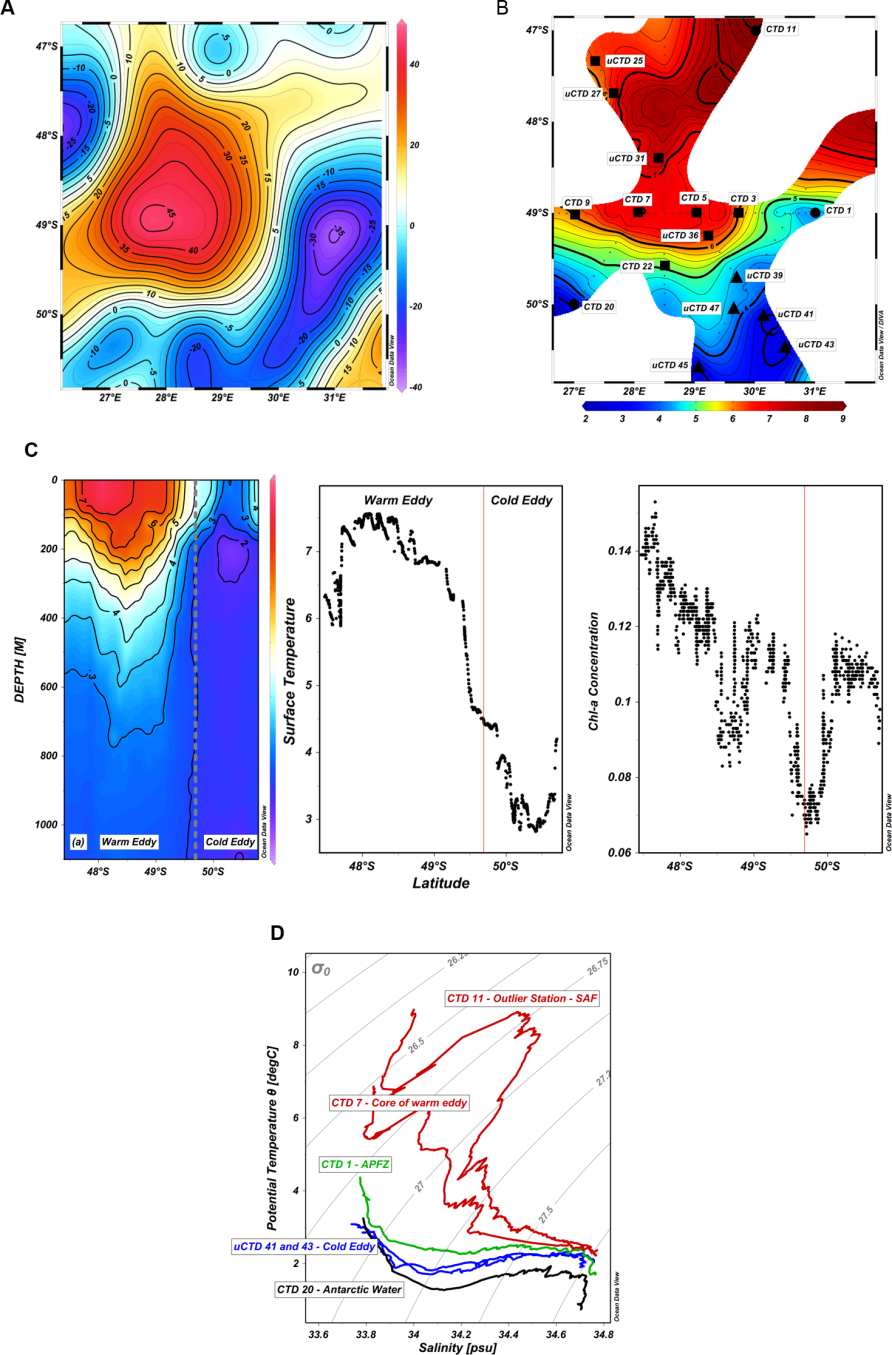
Physical characteristics of adjacent warm and cold core eddies. **(A)** Real Time of Merged Sea Level Anomalies (MSLA obtained from http://www.aviso.oceanobs.com*)* showing the position of the warm (positive anomaly) and cold core (negative anomaly) eddies. The positive anomaly is centred 28°E and 49°S, while a negative anomaly, presumed to be a cold Antarctic eddy was observed between 48.45°-51°S and 28.30°-32°E. **(B)** Sampling locations of adjacent cold- (▲) and warm-core eddies (■) situated within the Antarctic Polar Front Zone (APFZ) of Southern Ocean. Samples collected from the Subantarctic, APFZ and Antarctic domains are indicated (●). CTD (conductivity, temperature and depth) and uCTD (underway CTD) sampling stations are indicated on the map along with temperature data in ^o^C. Isotherms are at 0.25°C intervals. **(C)** Vertical cross section of temperature across both the warm and cold-core eddy. The grey dashed line demarcates the boundaries between the two features. Surface property plots across both warm and cold-core eddies (again separated by a red line), demonstrating surface temperature and Chl-a concentration (mg.m^-3^). **(D)** Temperature and salinity (T/S) profiles highlighting the cold-core eddy (blue), the core of the warm eddy and the Subantarctic Front (SAF) domain (red), the APFZ (green) and Antarctic waters (black). The profiles characterize the nature of both eddies in relation to the surrounding ocean environment.

Since water sampling was conducted within international waters, outside of the Prince Edward Islands Marine Protected Area, no sampling permits were required. In-situ oceanographic and biogeochemical data was collected between 22 April and 7 May 2012 onboard the South African research and supply vessel, the SA Agulhas I, as part of a survey of two contrasting eddies in the APFZ. Prior to the survey, two contrasting anomalies were identified from Real Time of Merged Sea Level Anomalies (MSLA- obtained from http://www.aviso.oceanobs.com) (Fig 2A) It was deduced via observed eddy properties that an intense sea surface height (SSH) anomaly (>40 cm) centred at 28°E and 49°S represented an anti-cyclonic eddy formed by the interaction of the Subantarctic Front (SAF) with the SWIR (Fig 2A and 2B). Lying directly southeast of this feature an intense negative anomaly (-35 cm), assumed to be a cold-core (Fig 2A) Antarctic eddy, was observed between 48°45’-50°30’S and 30°- 32°E. A total of 5 transects consisting of 21 CTD (conductivity, temperature and depth), 86 XBT (Expendable Bathythermograph)s, 17 UCTDs, 36 Stationary UCTD (Underway CTC) measurements and 30 biological stations were completed. Underway sampling was conducted using XBT and UCTD measurements. Sippican Deep-Blue XBTs were deployed to a maximum depth of 880 m, while the Ocean Science Sea-Bird UCTD was deployed to a maximum depth of 400 m with a 500 lb line measuring a vertical profile of salinity and temperature against depth. A full CTD study of the positive anomaly was undertaken to a maximum depth of 2000 m using a SBE 911plus (Sea-Bird Scientific). A rosette of 12 niskin bottles was deployed and bottles closed at standard depths of 2000 m, 1500 m, ~1200 m (O_2_ min), 1000 m, 500 m, 250 m, 75 m, 50 m, 20 m, 10 m, and 5 m. Samples were collected for salinity, nutrients (silicates, phosphates, nitrates and nitrites) and dissolved oxygen measurements. The salinity samples were processed with a Guildline model 8410A Portasal Salinometer to confirm the recent calibration of the CTD salinity sensor. Unfortunately at CTD station 22 the communications between the CTD rosette and deck unit failed and a contingency plan to replace the CTD stations with a vertical UCTD deployed in a stationary position to its maximum capable depth of 1200m was carried out for the remaining stations.

### Nutrients

During the eddy survey duplicate samples were taken at each CTD station and immediately frozen at -20°C. A total of 21 CTD stations were sampled. Nitrate and silicate concentrations were determined using an automated Flow Injection Analyzer system (Lachat Quickchem methods 31-114-27-1-D and 31-107-04-1-E) on return to Cape Town, while phosphate (PO_4_-P) and ambient nitrite (NO_2_-N) were determined manually using the spectrophotometric methods described in Grasshoff, Ehrhardt [26].

### Chlorophyll-a

Total integrated chlorophyll-a (chl-a) concentration at each station was determined from water samples collected at 5 standard depths (0, 5, 20, 50 and 100 m) using a rosette of Niskin bottles attached to the CTD. Chl-a concentration at each depth were determined fluorometrically. Chl-a samples were collected by filtering 250 ml of seawater through 25 mm glass fibre filters (Whatman GF/F). Chl-a was extracted from the filter with 7ml of 90% acetone over a 24 hr period at -20°C and pigment concentrations measured by fluorimetry as described previously [3].

### Genomic DNA extraction and 16S rRNA gene sequence amplification

Surface samples (5 m depth) were collected from different sites within the warm- and cold-core eddies (Fig 2B, Table 1). Two litres of seawater were initially filtered through 100 μm mesh to remove particulate matter, after which microbial biomass was collected by filtration through 0.22 μm membrane. The filters were immersed in RNALater (Qiagen) and stored at -20°C. Genomic DNA was extracted from half of the membrane by using the Power Water DNA Isolation Kit (MoBio, West Carlsbad, CA), according to the manufacturer’s instructions. The V4-V5 variable regions of the bacterial 16S rRNA gene were PCR-amplified, using the E517F (5′-CAG CAG CCG CGG TA A-3′) and E969-984R (5′-GTA AGG TTC YTCG CGT-3′) primers [27] which included multiplex identifier tags and template-specific nucleotides primers for each sample. PCR amplification was carried out using KAPAHiFi Hotstart DNA Polymerase (KAPA Biosystems) over 25 cycles using the conditions described in [28]. The reaction products were gel-purified using the Zymoclean™ Gel DNA Recovery Kit (Zymo Research), subjected to emulsion PCR, and then sequenced using the GS Junior Titanium Sequencer (454 Life Sciences, Roche). The sequence dataset has been submitted to the NCBI Sequence Read Archive (Accession number: SRP067732).

**Table 1:** Geographical location of all microbial sampling sites and associated physio-chemical characteristics. The location of each sample in relation to either the warm or cold eddy is shown in Fig 2B.

Sample	Longitude& Latitude	Eddy	Zone	Dissolved	Temp	Salinity		Nutrients	
				O_2_ (ml/L)	^o^C	(PSU)	SiO_4_ (μM)	PO_4_ (μM)	NO_3_+NO_2_ (μM)
**2012–01**	31°00’14”; 48°59’.96”	-	APFZ	6.87	4.37	33.774	8.05	2.965	21.46
**2012–11**	30°00’02”; 47°00’08”	-	SAF	6.18	8.97	34.008	1.70	1.256	14.82
**2012–20**	26°59’90”; 49°60’.00”	-	Antarctic	7.001	3.21	33.788	16.37	1.744	25.06
**2012–39**	29°46’45”; 49°46’.35”	Cold	Edge	ND	4.27	33.672	4.70	0.890	12.38
**2012–41**	30°08’59”; 50°06’.60”	Cold	Core	ND	3.08	33.741	12.19	1.484	14.22
**2012–43**	30°29’35”; 50°28’.24”	Cold	Core	ND	2.86	33.798	28.94	1.022	15.44
**2012–45**	29°07.00’; 50°36.00’	Cold	Core	ND	3.03	33.795	14.64	0.989	17.04
**2012–47**	29°38.00’; 50°04.00’	Cold	Edge*	ND	4.25	33.780	6.82	0.791	16.99
**2012–03**	30°00’50”; 48°59’88”	Warm	Edge*	6.71	5.45	33.800	4.93	1.465	23.15
**2012–36**	29°14’13”; 49°15’41”	Warm	Edge*	ND	6.28	33.736	5.35	1.121	17.68
**2012–07**	28°00’09”; 49°00’19”	Warm	Core	6.544	6.85	33.831	3.57	1.535	25.22
**2012–05**	29°00’47”; 48°59z’98”	Warm	Middle	6.466	6.87	33.777	3.59	1.919	24.00
**2012–25**	28°30’00”; 48°20’00”	Warm	Middle	ND	7.48	33.701	2.00	1.022	15.55
**2012–27**	27°37’42”; 47°41’42”	Warm	Middle	ND	6.26	33.698	2.14	0.75	12.98
**2012–31**	28°22’09”; 48°22’21”	Warm	Middle	ND	7.06	33.809	1.24	0.626	9.96
**2012–09**	27°00’08”; 49°00’02”	Warm	Edge	8.020	8.67	33.808	5.19	1.605	28.55
**2012–22**	28°29’99”; 49°35’05”	Warm	Edge	6.715	5.077	33.780	6.39	1.484	20.30

APFZ” Antarctic Polar Front Zone; SAF: Subantarctic Front; ND: no data available

*: edge between the two eddies

### Pyrosequencing data analysis

A total of 118510 raw 454 sequence reads with an average length of 400 nts were quality-filtered and cured of primer/tag sequences using the standard software provided by Roche 454 Life Sciences. The Mothur software package (v 1.34.4) [29] was used to remove sequences of < 200 nts, reads that contained ambiguous nucleotides and homopolymeric runs of >7 from the dataset.Mitochondrial sequences were also excluded from the analysis and chimeric sequences were removed subsequent to identification using the UChime algorithm [30]. A summary of the dataset used in this study can be found in the supplementary data (S1 Table). The sequence data sets were aligned against the SILVA 16S rRNA gene database version 123 [31] and a pairwise distance matrix was created from the curated aligned datasets to group sequences into Operational Taxonomic Units (OTUs) at a confidence level of 97%. Classification of the reads were done to the genus level using the Naïve Bayesian classifier algorithm [32, 33] against the SILVA 16S rRNA gene database with a confidence threshold of 80% to assign taxonomic identity.

All the statistical analyses were performed in R statistical package v.3.2.0 (http://www.r-project.org/). The Vegan package [34] in R was used to generate a non-metric Multiple Dimension Scaling (nMDS) analyses as well as identify significant differences between the two eddy systems (ANOSIM) [35]. Dominant bacterial OTUs were further compared to the nucleotide database hosted at GenBank by using NCBI-BLAST tool [36] and EzTaxon-e server based BLAST analysis [37] and the results are provided in the supplementary data (S2 Table). Microphytoplankton diversity was analyzed using the chloroplast OTUs in the sequence datasets. Dominant OTUs were identified using the PhytoREF reference database [38] and the results were tabulated (S3 Table).

## Results

### Physical characteristics of adjacent warm- and cold-core eddies

**Fig 3:**
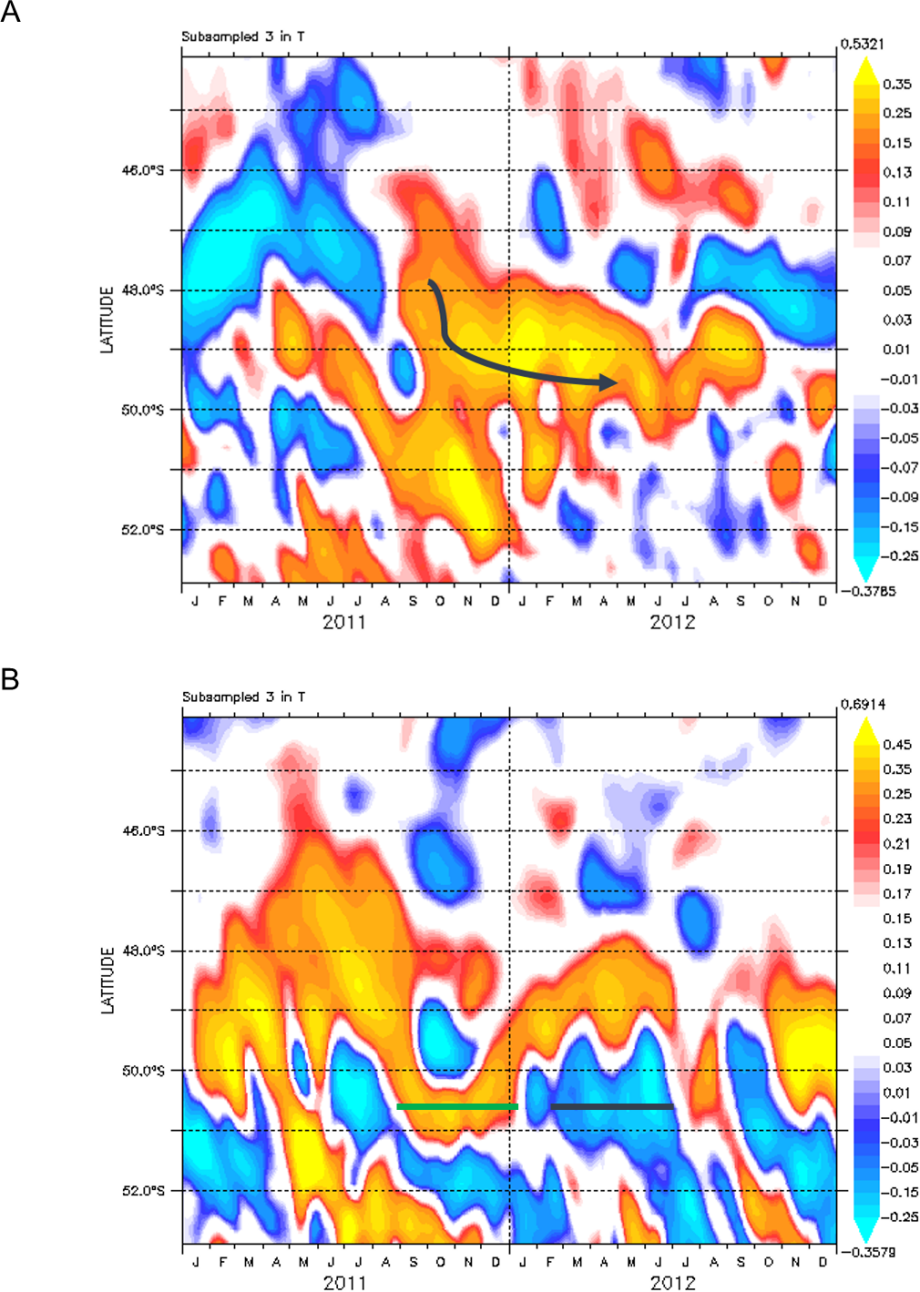
Time series of altimetry data showing the development of the warm- and cold-core eddies. **(A)** Hofmöller plot of altimetry data at 27°E (in m) showing the warm-core eddy between September 2011 and at the time of the April 2012 survey. The arrow represents the southward path taken by the eddy during this period. **(B)** Hofmöller plot of altimetry data at 29°E (in m) showing the cold-core eddy (black line) from its time of formation in March 2012 to the time of the survey (April-May 2012). The cold eddy is considered to be juvenile, having been formed in 6 weeks prior to the voyage and intensifying until June 2012. Prior to the formation of the cold eddy, a positive anomaly indicative of a warm eddy (represented by the green line) is evident at 51°S.

A positive anomaly observed during the cruise in 2012 from altimetry data and centred at 28°E and 49°S (Fig 2A), was assumed to represent a warm-core eddy lying directly within the region of highest SSH variability (Fig 1). Its diameter, determined from both altimetry and hydrographic observations (Fig 2A and 2B), was approximately 215 km and CTD temperature data showed that the anomaly extended to at least 800 m in depth (Fig 2C). Underway sea surface temperatures within the core of the anomaly ranged between 7–7.4°C (Fig 2C), dissolved oxygen from 6.8–6.4 ml/L and surface salinity averaged 33.7 PSU, which relative to its surroundings, confirmed that the anomaly was a warm-core eddy. Geostrophic velocities (data not shown) inferred from altimetry (Fig 2A) exceeded 0.65 m s^-1^ at the edge of this feature providing further support that the anomaly observed was anti-cyclonic in its rotation. It was assumed, given previous investigations in this region [3] that this feature had become detached from the SAF to the north. Stations occupied in the core of the warm eddy recording a subsurface salinity maximum of 34.608 PSU at σ_0_ 26.78 (Fig 2D), confirmed the presence of Subantarctic Surface Water, which had become modified during its translocation southwards into the APFZ (Fig 2D). Indeed, a time series of altimetry data for two years (Fig 3A) confirmed that the eddy was in fact mature, having been formed in September 2011 and relocated southwards to 49°S at the time of the April 2012 survey. In addition, comparisons with a station located north of the eddy and within the proper SAF domain at 47°S, and thus typical of unmodified SASW, showed a similarity with that of the core of this feature (34.476 PSU at σ_0_26.739). Although water masses within this eddy were colder (~2°C) and fresher (~0.2 PSU) than within the SAF domain proper (Table 2) the similarity brought on by the salinity subsurface maximum (between 140–150 m) in profile was clear and further confirmed that this feature consisted of SASW that had become modified over time and distance south (Fig 2D).

**Table 2:** Comparison across the warm-core (Subantarctic) and cold-core (Antarctic) eddies during the 2012 survey.

	Cold-core eddy			Warm-core eddy			APFZ
	Centre	Northern Edge	Southern Edge	Centre	Northern Edge	Southern Edge	
Latitude	50° 16’S	49°48’S	50° 42’S	48°42’S	47°42’S	49°16’S	47°33’S
Temperature (°C)	3.01	4.38	4.19	7.18	6.02	6.33	6.8
[Chl-a] (mg.m-3)	0.115	0.0813	0.09	0.083	0.13	0.11	0.083

APFZ: Antarctic Polar Front Zone, included for comparison with eddy

A negative anomaly, was observed from altimetry data south-east of the warm-core eddy between 48°45’-50°30’S and 30–32°E (Fig 2A). Its diameter, from both altimetry and hydrographic observations (Fig 2A and 2B) was approximately 200 km and extended to depths of 800 m. Steep shoaling of isotherms and isohalines, which are characteristic of a cyclonic eddy, were apparent in the upper layers and a well-developed subsurface temperature minimum layer (T_min_ ~1.66°C), capped in the top 100 m by a relatively warm <3°C layer and fresh <33.7 PSU, was observed (Fig 2C). South of the APF, Antarctic Surface Water in summer is characterized by a shallow T_min_ associated with the remnants of Winter Water—a previous winter mixed layer capped by seasonal warming and freshening [39]. This Winter Water was observed between 150–170 m and ranged in a T_min_ of 1.74–1.89°C. Of interest is a comparison between the older, warm-core eddy (Fig 3A) formed in 2011and the altimetry time series for the cold eddy (Fig 3B), which confirmed that the cold-core eddy had been recently formed in early March 2012 and within the Antarctic belt. Indeed, subsurface water masses between 200–500 m (Fig 2C and 2D) within the core of the eddy were typically Antarctic, displaying physical characteristics <2°C cooler and >0.4 PSU saltier than the surrounding APFZ waters, again indicative that the eddy had originated from south of the APF (Fig 2C and 2D). There is little doubt that both eddies had their origins in the Antarctic and Subantarctic zones, having been shed from the APF and SAF a number of months previously. These two contra-rotating eddies provided a fortuitous opportunity to study, for the first time, the importance of eddies, from these different oceanic environments and with contrasting internal dynamics (upwelling vs downwelling) on the entrainment of microbes within the APFZ and the mechanisms responsible for their distribution.

### Nutrient analysis and primary productivity

The APFZ in which these eddies were observed, is characterized as a zone of high-nutrient, low-chlorophyll [40, 41] due to light limitation, deficiency of trace nutrients such as iron, silica limitation of diatom production [42] and grazing by zooplankton [43]. High levels of chlorophyll concentration (Fig 2C) corresponded to the upwelling regions within both eddies i.e. centre of the cold eddy (0.115 mg.m^-3^) and at the edge of the warm eddy (0.11–0.13 mg.m^-3^, Table 2). Silicate concentrations were significantly elevated in the cold-core eddy with the highest levels recorded in zones of upwelling (12.19–28.94 μM in the core) vs the warm-core eddy (4.63–6.39 μM at the edges). P and N levels were also highest in zones of upwelling in both eddies, but overall, higher in the warm-core eddy (Table 1). Primary productivity, as a function of Chl-a concentration, was also highest in the zones of upwelling in both eddies with the highest levels recorded in the core of the cold-core eddy (Table 2). Together, the physical and biogeochemical data demonstrated that the adjacent features exhibited the typical characteristics of warm- and cold-core eddies originating from the Subantarctic and Antarctic, respectively. This provided a unique opportunity to investigate the microbial communities in eddies from different oceanic environments, and with contrasting internal dynamics (upwelling vs downwelling), and their potential influence on ecosystem function of the APFZ.

### Bacterial community analysis

**Fig 4:**
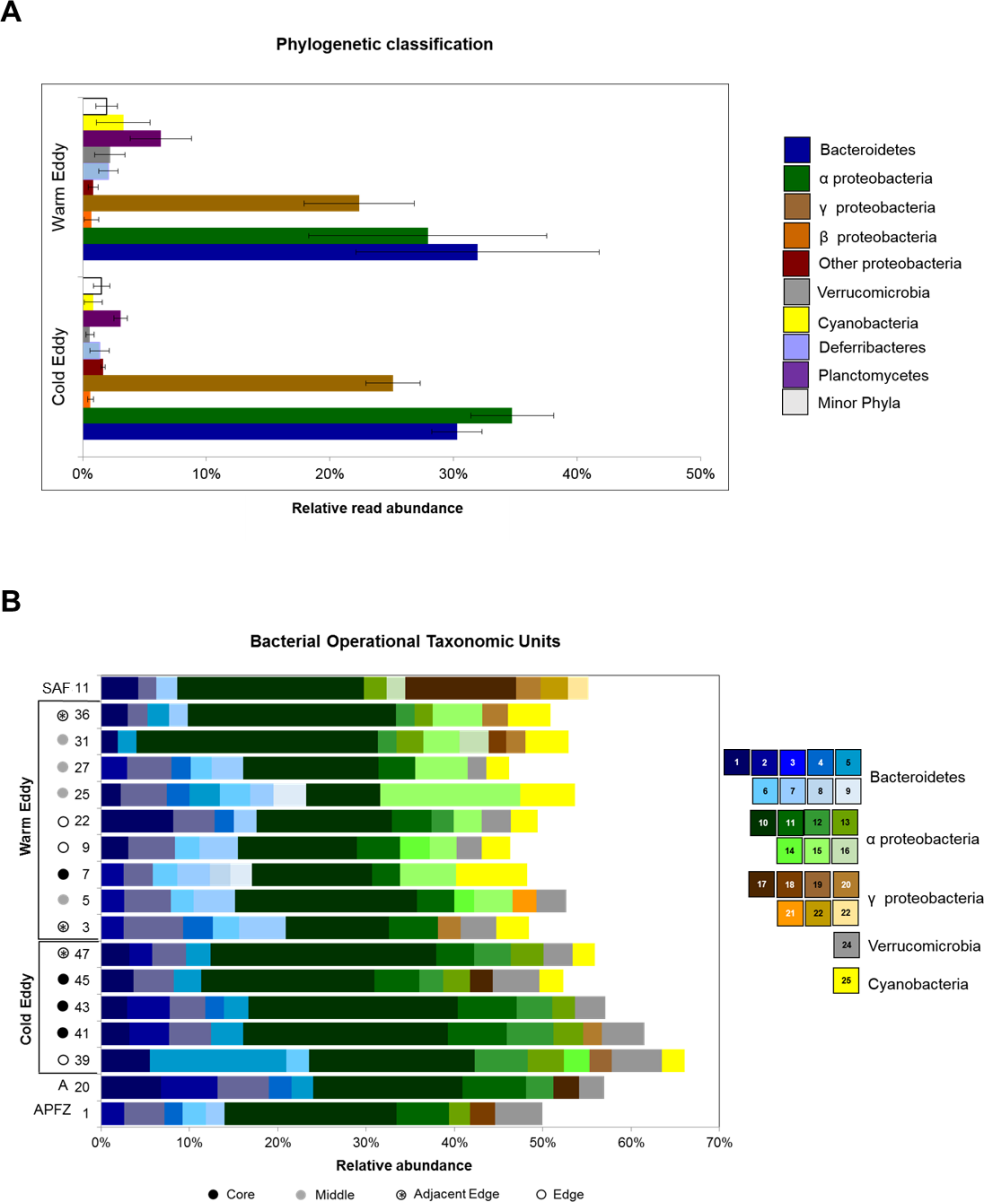
Bacterial diversity and community structure in the Southern Ocean eddies. **(A)** Phylogenetic classification of bacterial taxa. The data is expressed as the relative percentage of phyla and subphyla present within the cold eddy (n = 5 samples), warm eddy (n = 9 samples). Error bar represents standard error mean between samples. **(B)** Relative abundance of dominant bacterial species (B_OTUs). The bacterial 16S rRNA datasets were classified at the species level (OTU_0.03_) with the Silva reference database (Version 123) using the Mothur platform. SAF: Subantarctic Front; APFZ: Antarctic Polar Frontal Zone; A: Antarctic.

A total of 90402 bacterial 16S rRNA sequence reads, obtained from samples collected across the two eddies, were analyzed (S1 Table). The Proteobacteria phylum, comprising mainly alpha- and gammaproteobacteria, accounted for almost half of the total number of reads, while approximately 25% of the reads were classified as Bacteroidetes taxa. Also relatively abundant were reads classified within the Planctomycetes, Cyanobacteria and Verrucomicrobia phyla, with Planctomycetes and Cyanobacteria appearing to be more abundant in the warm-core eddy relative to the cold ore eddy (Fig 4A). The bacterial communities of both eddies was dominated by 25 OTUs, representing between 50–65% of the total reads from each sample (Fig 4B). Unsurprisingly the alphaproteobacterium, *Pelagibacter ubique* (B_OTU10), was the most abundant OTU in all samples, as were B_OTU11, B_OTTU12 (Alphaproteobacteria) and B_OTU3 (Bacteroidetes). B_OTU7 and B_OTU15, identified as bacteroidetes and alphaproteobacterial species, respectively, were present only in the warm-core eddy. While B_OTU6 (Flavibacteria) had a similar distribution (Fig 4B), it was also detected in a sample collected from the edge of the cold-core eddy (CTD39). The presence of these three OTUs in the Subantarctic sample (CTD11) is consistent with their entrainment by the warm-core eddy when it was shed from the Subantarctic Front several months earlier (Fig 3A). A cyanobacterial OTU, B_OTU14, was significantly more abundant in the warm-core eddy than in the cold-core eddy and absent in the samples collected from Subantarctic, APFZ and Antarctic waters (Fig 4B).

### Characterization of microphytoplankton diversity

**Fig 5:**
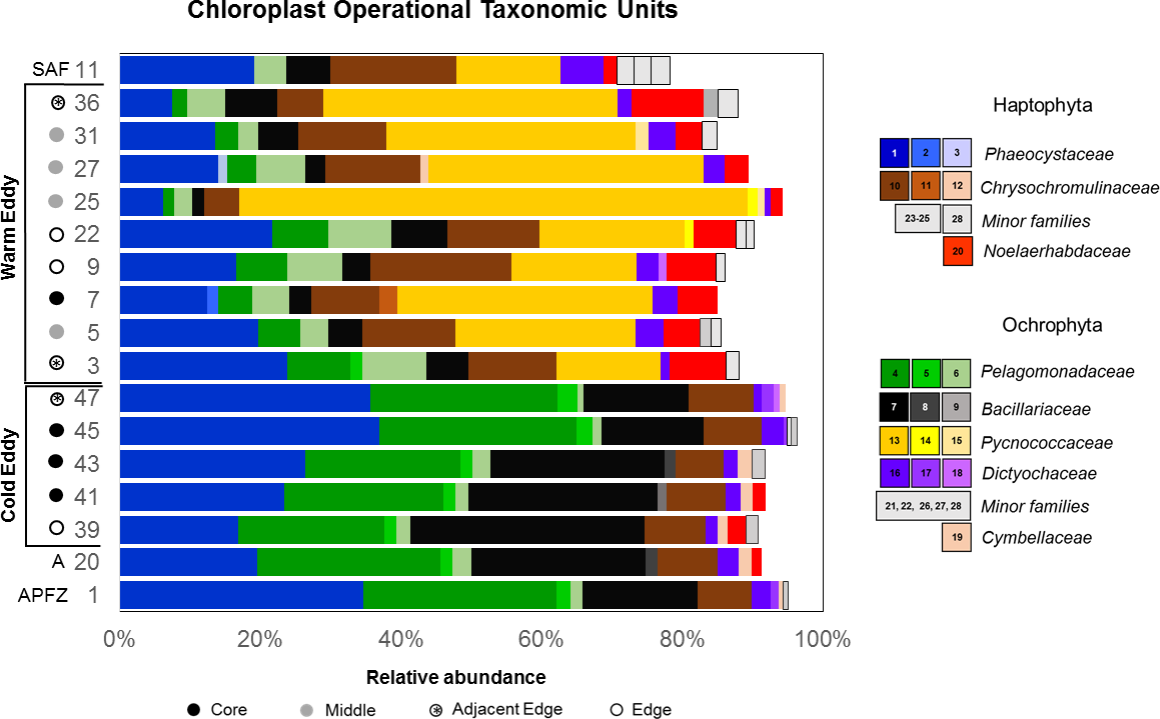
Microphytoplankton diversity in the Southern Ocean eddies. Chloroplast OTUs (C_OTUs) were extracted from 16S sequence datasets and the relative abundance of the top 10 dominant C_OTUs in each sample are represented in the bar graph. C_OTUs were identified using the PhytoREF reference database. SAF: Subantarctic Front; APFZ: Antarctic Polar Frontal Zone; A: Antarctic.

The primers used to amplify the bacterial 16S rRNA gene sequence also amplify the V4-V5 region from chloroplast DNA [44] and a total of 14393 chloroplast sequence reads were subjected to an OTU analysis. As with the bacteria, the microphytoplankton communities across all the samples were dominated by 29 OTUs, belonging to two major phyla (Haptophyta and Ochrophyta) that accounted for between 80–95% of all the chloroplast reads obtained (Fig 5). There were clear differences between the microplankton communities of the two eddies, with the Ochrophyte, C_OTU13 (Family *Pycnococcaceae*), being the dominant species in the warm-core eddy and Subantarctic waters, but absent in the cold-core eddy, APFZ and Antarctic waters. C_OTU4 (Family *Pelagomonadaceae*) and C_OTU7 (*Pseudo-nitzschia seriata*) were most abundant in the cold-core eddy, Antarctic and APFZ waters, while C_OTU6, (*Pelagomonas calceolata*) was more abundant in the warm-core eddy and Subantarctic. With respect to the Haplophyta, C_OTU10 (*Chrysochromulina throndsenii*) and C_OTU20 (*Emiliania huxleyi*) were most abundant in the warm-core eddy while C_OTU11 (Family *Chrysochromulinaceae*) predominated in the cold-core eddy (Fig 5). As with the bacterial communities, the presence of eddy-specific chloroplast OTUs that were also present in the waters from which these eddies originated is consistent with the entrainment of biota from their point of origin.

### Distinct microbial communities in warm- versus cold-core eddies

**Fig 6:**
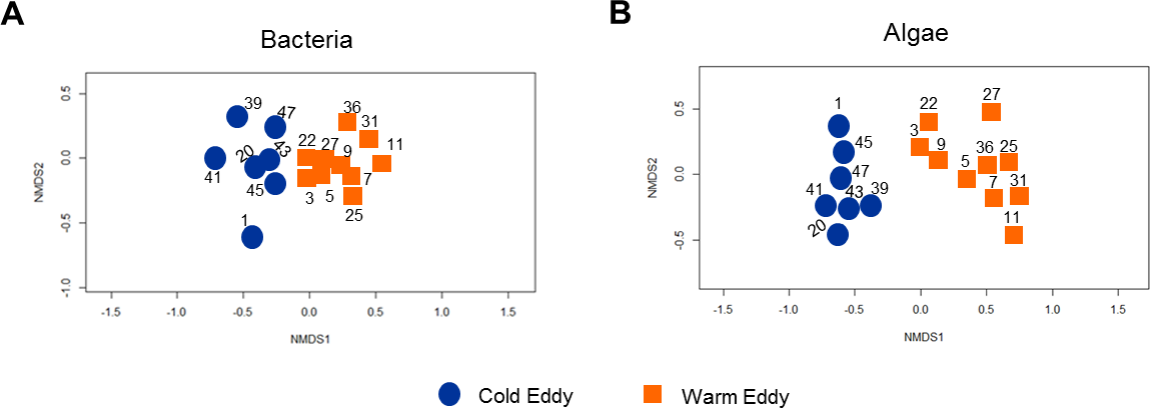
Non-metric Multidimensional Scaling (nMDS) ordination of microbial communities in cold- vs warm-core Southern Ocean eddies. NMDS plots of bacterial **(A)** and algal **(B)** communities were based on a Bray–Curtis dissimilarity matrix (stress = 0.984). The ordination plots were derived from the 16S rRNA bacterial and chloroplast OTUs classified at the species (97%) level using the Mothur software platform. The significance of the dissimilarity between microbial communities was statistically confirmed (p = <0.05) using the Analysis of Similarity (ANOSIM) test using the Vegan package in the R platform.

A Non-metric Multiple Dimension Scaling (NMDS) analysis was conducted to examine the relatedness between microbial communities present in the two eddies. The scatter plot analysis showed that sequence datasets for both the bacterial and microphytoplankton communities clustered separately into two distinct groups representing each of the two eddies (Fig 6). These findings were statistically confirmed by analyzing significance between bacterial (ANOVA, R = 0.6493, *P* < 0.001) and the microphytoplankton communities (ANOVA, R = 0.8171, *P* < 0.0009) in samples collected from the warm and cold-core eddies (S4 Table). The NMDS analysis confirmed the close relationship between the microbial communities in the warm-core eddy and the Subantarctic (CTD11), while the Antarctic sample (CTD20) clustered within the cold-core eddy communities (Fig 6). Interestingly the microphytoplankton communities in the APFZ (CTD01) and those of the cold-core eddy were closely related (Fig 5), but there was a significant difference with respect to bacterial communities (Fig 6A, S4 Table).

## Discussion

Direct measurements near the Drake Passage, south of Tasmania and south of New Zealand [11, 45, 46] show consistent eddy advection across the ACC, supporting a global estimate derived from satellite altimetry data. The geographic distribution of regions exhibiting high levels of eddy kinetic energy in the Southern Ocean are, however, very inhomogeneous. Meridional exchanges in the Southern Ocean are a key component for the accurate simulation of the coupled global ocean-biological models. If the assumption is correct that a part of this exchange comes about through mesoscale eddies, then the quantification at regions where mesoscale turbulence is high may be crucial. Our initial focus was on the mature warm-core eddy spawned from the Subantarctic domain in August 2011 (Fig 3A), because this type of eddy is yet to be biologically characterized. The appearance of an adjacent and recently spawned cold-core eddy that originated during April 2012 from Antarctic waters south of the APF, provided the rare opportunity for a comparative observational study. In this study we demonstrate that eddies are responsible for the redistribution and local variations in microbial communities across the APF.

The microbial communities entrained by the two eddy types are quite distinct. The bacterial communities in both eddies were dominated by Proteobacteria and Bacteroidetes, with Cyanobacteria and Planctomycetes being more abundant in the warm-core eddy. Interestingly, the cyanobacterial reads belonged to a single, dominant OTU, while the absence of dominant Planctomycetes OTUs infers more species richness compared to the other phyla. Given the fairly even distribution of the dominant OTUs between these two eddies, it is likely that Planctomycetes diversity may be an important factor in distinguishing the bacterial communities of the two eddies. By contrast there were striking differences between the dominant microphytoplankton OTUs in the two eddies that correspond with the waters from which they were spawned. Overall, the close relationship between bacterial and microphytoplankton communities in the warm- and cold-core eddies with the Subantarctic and Antarctic, respectively, are consistent with the entrainment of biota from the eddy source, transport to and eventual release into the APFZ.

Upwelling of nutrient-rich bottom waters by cold-core eddies results in high levels of primary productivity that make them important foraging grounds for top predators [47]. While there were differences in the overall nutrient concentrations in the two eddies, most notably elevated levels of silicates in the cold-core eddy, we did not observed a marked difference in primary productivity (as a function of chl-a concentration). Oligotrophic waters are typically dominated by cyanobacteria, whereas microphytoplankton are dominant in regions of upwelling [48]. Thus the observed increase in the abundance of cyanobacteria may indicate oligotrophic conditions in the warm-core eddy.

This is the first investigation of eddy influence on the microbial exchange at the SWIR and the study emphasizes the importance of understanding the coupling of physics to microbial ecology on a mesoscale level. Our data suggest that the formation, and in particular, the translocation of warm- and cold-core eddies at the SWIR may play a significant role in the redistribution of microbial diversity across the APFZ. How many of these eddies are formed each year remains unknown so it is important that further quantitative studies projecting this region on a circumpolar footing are undertaken in order to establish the importance of the SWIR as a microbial diversity hotspot. The nature of eddy fluxes across the ACC may be sensitive to current changes in the velocity and position of the ACC. Subsurface observations have shown an increase of 0.17°C since 1950 in the Southern Ocean, possibly brought on by a southward shift in the position of the ACC [49]. We speculate that these changes are not independent and that variations in the wind stress can induce changes in the intensity of the Southern Ocean eddy field. In short, an increase in the poleward eddy flux can be attributed to the strengthening westerly wind belt [50]. Closer examination of Gille’s dataset identifies that these trends occur directly south of each band of high eddy kinetic energy–the SWIR being one such example. Changes in the intensity and geographic position of the ACC and consequently its eddy field are likely to coincide with dramatic shifts in the biogeochemical uptake through eddy activity and advection within the Southern Ocean and in particular within the Antarctic Polar Frontal Zone.

Despite their ecological importance, there is surprisingly little data on the microbial communities in the open ocean waters of the Southern Ocean and the ACC. Global marine microbiome projects such as the Tara project [51] focused on the Northern hemisphere and sub-tropical regions, while other studies have concentrated on the coastal marine areas of high productivity [52, 53]. While our study sheds some light on microbial community dynamics within the APFZ, there is an acknowledged need for a comprehensive mapping of the microbial communities in the open waters of the Southern Ocean.

## Supporting information

S1 Table Summary of 454 sequence data used in this study. (PDF)

S2 Table Dominant bacterial OTUs and their closest phylogenetic neighbors. (PDF)

S3 Table Dominant phytoplankton OTUs and their nearest phylogenetic neighbors. (PDF)

S4 Table Statistical analysis of differences between microbial communities in warm-and col d-core eddies. (PDF)
