# Supplementary material for: A pivotal role for ocean eddies in the distribution of microbial communities across the Antarctic Circumpolar Current
Source: PLoS One. 2017 Aug 21;12(8):e0183400. doi: 10.1371/journal.pone.0183400 (PMC5565106; doi:10.1371/journal.pone.0183400)
Supplement: S1 Table — (PDF) [file pone.0183400.s001.pdf]

S1 Table: Summary of 454 sequence data used in this study

Sample	Raw Sequences	Curated Sequences		Bacterial OTUs		Chloroplast OTUs	
		Bacterial	Chloroplast	Total	- singletons	Total	- singletons
2012-01	7210	5303	818	316	200	27	19
2012-11	8861	7210	653	354	171	61	39
2012-20	3911	3348	487	186	114	31	19
2012-39	7974	5296	1788	266	166	42	27
2012-41	3836	3105	648	201	119	34	21
2012-43	9350	8084	1054	287	181	37	24
2012-45	4299	3624	524	230	144	23	15
2012-47	6265	5166	481	268	151	24	18
2012-03	4853	3813	645	217	147	37	26
2012-36	9935	6658	893	351	190	50	23
2012-07	6062	4365	759	278	155	51	33
2012-05	9043	6984	1192	340	185	58	34
2012-25	7684	6322	1388	277	170	38	30
2012-27	7287	5887	895	302	183	50	28
2012-31	9143	6166	708	359	216	51	31
2012-09	4974	3820	651	233	145	40	27
2012-22	7823	5251	809	248	165	38	26

Total raw sequence reads: 118510: Curated bacterial reads: 90402; Curated Chloroplast reads:
