# Supplementary material for: A pivotal role for ocean eddies in the distribution of microbial communities across the Antarctic Circumpolar Current
Source: PLoS One. 2017 Aug 21;12(8):e0183400. doi: 10.1371/journal.pone.0183400 (PMC5565106; doi:10.1371/journal.pone.0183400)
Supplement: S2 Table — (PDF) [file pone.0183400.s002.pdf]

S2 Table: Dominant bacterial OTUs and their closest phylogenetic neighbors

Bacterial OTU	Nearest Neighbor (NCBI-BLAST)	Similarity (%)	EZ taxon Blast	Similarity (%)	Phylum	Isolation source
B_OTU1	*Lacinutrix algicola* AKS293 (NR043592)	92	*Lacinutrix algicola* AKS293^T	92	Bacteroidetes	Sea Water, Korea
B_OTU2	*Owenweeksia hongkongensis* UST20020801^T (NR040990)	92	*Flavobacteria* class	99	Bacteroidetes	Southern Ocean
B_OTU3	*Yeosuana aromativorans* GW1-1^T (NR043102)	92	*Flavobacteria* class	98.63	Bacteroidetes	South Sea, Korea
B_OTU4	*Corallibacter vietnamensis* KMM 6217^T (NR122083)	98	*Flavobacteria* class	100	Bacteroidetes	Sea Water
B_OTU5	Tenacibaculum ovolyticum NBRC 15947 (NR113826)	99	*Tenacibaculum ovolyticum* IFO 15947*T*	100	Bacteroidetes	Sea Water, Japan
B_OTU6	*Aquibacter zeaxanthinifaciens* CC-AMZ-304^T (NR125631)	93	*Flavobacteria* class	99	Bacteroidetes	Surface Sea Water
B_OTU7	*Lacinutrix algicola* AKS293^T (NR043592)	92	*Lacinutrix algicola* AKS293^T (DQ167238)	92	Bacteroidetes	Sea Water, Korea
B_OTU8	*Owenweeksia hongkongensis* UST20020801^T (NR040990)	92	*Flavobacteria* class	99	Bacteroidetes	Southern Ocean
B_OTU9	*Formosa arctica* IMCC9485^T (NR125695)	99	*Formosa* sp.	98	Bacteroidetes	Arctic seawater
B_OTU10	*Pelagibacter ubique* HTCC1062^T (NR074224)	100	*Pelagibacter ubique*	100	Alphaproteobacteria	Arctic Ocean
B_OTU11	*Vesicomyosocius okutanii* HA^T (NR074939)	97	*Candidatus* sp.	96	Alphaproteobacteria	Southern Ocean
B_OTU12	*Pelagibacter ubique* HTCC1062^T (NR074224)	93	Uncultured bacterium	98	Alphaproteobacteria	Arctic Ocean
B_OTU13	*Pelagibacter ubique* HTCC1062^T (NR074224)	93	Uncultured bacterium	99	Alphaproteobacteria	Red Sea
B_OTU14	*Brasilonema tolantongensis* Tolantongo^T (NR118308)	87	Uncultured bacterium	100	Alphaproteobacteria	Sea Water
B_OTU15	*Halospirulina tapeticola* CCC Baja-95^T (NR026510)	83	Uncultured bacterium	100	Alphaproteobacteria	Sea Water
B_OTU16	*Rhodovulum euryhalinum* DSM	87	Uncultured bacterium	99	Alphaproteobacteria	Seawater pools

	4868T (NR043406)					
B_OTU17	*Pseudoalteromonas undina* NBRC 103039T (NR114191)	100	*Pseudoalteromonas* sp.	99	Gammaproteobacteria	Sea Water
B_OTU18	*Acinetobacter johnsonii* ATCC 17909T (NR117624)	100	*Acinetobacter johnsonii* CIP 64.6T	100	Gammaproteobacteria	Sea Water
B_OTU19)	*Haliea mediterranea* 7SM29T (NR116976)	97	Uncultured bacterium	100	Gammaproteobacteria	Southern Ocean
B_OTU20	*Pseudomonas mucidolens* NBRC 103159T (NR114225)	90	Uncultured bacterium	99.32	Gammaproteobacteria	Southern Ocean
B_OTU21	*Cycloclasticus spirillensus* M4-6T (NR115117)	89	Uncultured bacterium	99.08	Gammaproteobacteria	Marine sediments
B_OTU22	*Acinetobacter marinus* SW-3T (NR115298)	83	Uncultured bacterium	100	Gammaproteobacteria	Yellow Sea, Korea
B_OTU23	*Oleispira antarctica* RB-8T (NR025522)	100	*Oleispira antarctica* RB-8T	100	Gammaproteobacteria	Antarctic coastal sea water
B_OTU24	*Roseibacillus ishigakijimensis* MN1-741T (NR041621)	93	Uncultured bacterium	99.8	Verrucomicrobia	Sea Water, Japan
B_OTU25	*Chamaesiphon minutus* PCC 6605T (NR102459)	86	Uncultured bacterium	100	Cyanobacteria	Sea Water
