# Supplementary material for: A pivotal role for ocean eddies in the distribution of microbial communities across the Antarctic Circumpolar Current
Source: PLoS One. 2017 Aug 21;12(8):e0183400. doi: 10.1371/journal.pone.0183400 (PMC5565106; doi:10.1371/journal.pone.0183400)
Supplement: S3 Table — (PDF) [file pone.0183400.s003.pdf]

S3 Table: Dominant phytoplankton OTUs and their nearest phylogenetic neighbors

Chloroplast OTU	Nearest Neighbour (BLAST)	BLAST score %	Phylum	Family	Source
C_OTU1	Uncultured *Phaeocystis* clone ST4_13 (KT956298)	100	Haplophyta	*Phaeocystaceae*	Coastal Antarctic waters
C_OTU2	Uncultured haptophyte clone MC615-75 (EF052146)	94	Haplophyta	*Phaeocystaceae*	Indian Ocean
C_OTU3	Uncultured haptophyte clone MC622-67 16S (EF052227)	99	Haplophyta	*Phaeocystaceae*	northeast Atlantic Ocean
C_OTU4	Uncultured *Pelagomonas* clone ST4_40(KT956315)	99	Ochrophyta	*Pelagomonadaceae*	Coastal Antarctic waters
C_OTU5	Uncultured bacterium clone HglApr728 (JX016141)	100	Ochrophyta	*Pelagomonadaceae*	Antarctic waters
C_OTU6	*Pelagomonas calceolata* chloroplast (LN735508)	100	Ochrophyta	*Pelagomonadaceae*	Antarctic waters
C_OTU7	*Pseudo-nitzschia seriata* isolate C98 c	100	Ochrophyta	*Bacillariaceae* (diatoms)	Arctic waters
C_OTU8	unidentified marine bacterioplankton (KC001703)	99	Ochrophyta	*Bacillariaceae* (diatoms)	Antarctic waters
C_OTU9	*Virgulinella fragilis* clone J501-203 (JN207212)	97	Ochrophyta	*Bacillariaceae* (diatoms)	Pacific region
C_OTU10	*Chrysochromulina throndsenii* chloroplast (LN735331)	100	Haplophyta	*Chrysochromulinaceae*	Atlantic Ocean:North Sea
C_OTU11	Uncultured eukaryote clone Ellett_EG3 (GQ863883)	97	Haplophyta	*Chrysochromulinaceae*	Antarctic waters
C_OTU12	Uncultured phototrophic Solas16400A-NG08 (FJ797589)	98	Haplophyta	*Chrysochromulinaceae*	northeast Atlantic Ocean
C_OTU13	chloroplast uncultured phototrophic eukaryote (FJ649255)	100	Ochrophyta	*Pycnococcaceae*	South East Pacific Ocean
C_OTU14	Uncultured bacterium clone SeaWat_24395 (JQ196073)	99	Ochrophyta	*Pycnococcaceae*	Arctic Ocean
C_OTU15	Uncultured eukaryote clone Ellett_EG3 (GQ863883)	99	Ochrophyta	*Pycnococcaceae*	North Atlantic
C_OTU16	Uncultured diatom clone PEACE2006/111_P3 (EU394568)	100	Ochrophyta	*Dictyochaceae*	Atlantic Ocean:North Sea
C_OTU17	Uncultured picoeukaryote clone	100	Ochrophyta	*Dictyochaceae*	Indian Ocean

	7656BH921_SP6 (JX291757)				
C_OTU18	Uncultured diatom clone GOM_WB8-19 (GQ250620)	94	Ochrophyta	*Dictyochaceae*	Gulf of Mexico
C_OTU19	chloroplast uncultured phototrophic eukaryote (EU005678)	100	Ochrophyta	*Cymbellaceae*	Antarctic waters
C_OTU20	*Emiliania huxleyi* chloroplast (LN735407)	100	Haplophyta	*Noelaerhabdaceae*	Atlantic Ocean:North Sea
C_OTU21	*Micromonas pusilla* chloroplast (LN735344)	99	Ochrophyta	*Mamiellaceae*	Arctic Ocean
C_OTU22	chloroplast *Micromonas pusilla* (LN735276)	100	Ochrophyta	*Mamiellaceae*	Antarctic waters
C_OTU23	plastid uncultured haptophyte (EF052062)	100	Haplophyta	*Prymnesiaceae* (Coccolithophores)	Atlantic Ocean
C_OTU24	Uncultured bacterium clone U1369-177 (JN986188)	100	Haplophyta	*Prymnesiaceae* (Coccolithophores)	Antarctic waters
C_OTU25	eukaryote clone Ellett_EG3_10m (GQ863883)	94	Haplophyta	*Prymnesiaceae* (Coccolithophores)	North Atlantic
C_OTU26	*Corethron pennatum* chloroplast 16S (AJ536466)	100	Ochrophyta	*Corethraceae*	Indian Ocean
