# Supplementary material for: A pivotal role for ocean eddies in the distribution of microbial communities across the Antarctic Circumpolar Current
Source: PLoS One. 2017 Aug 21;12(8):e0183400. doi: 10.1371/journal.pone.0183400 (PMC5565106; doi:10.1371/journal.pone.0183400)
Supplement: S4 Table — (PDF) [file pone.0183400.s004.pdf]

S4 Table: Statistical analysis of differences between microbial communities in warm-and col d-core eddies

	**Bacterial OTUs**			**Chloroplast OTUs**		
	**R value**	**P Value**	**Difference**	**R value**	**P Value**	**Difference**
Cold- vs. warm eddy	0.6493	0.001	Significant	0.8171	0.0009	Significant
APFZ vs. cold eddy	0.4671	0.0009	Significant	-0.28	0.69	None
SAF vs. warm eddy	-0.04	0.46	None	0.2963	0.11	None
